# What factors interfere with the performance of preschool children in the language subtest of Bayley-III?

**DOI:** 10.1590/2317-1782/20212020200

**Published:** 2021-11-01

**Authors:** Antonio Marcos Oliveira de Lima, Ana Manhani Cáceres-Assenço

**Affiliations:** 1 Laboratório de Desenvolvimento da Linguagem, Departamento de Fonoaudiologia, Centro de Ciências da Saúde, Universidade Federal do Rio Grande do Norte – UFRN - Natal (RN), Brasil.

**Keywords:** Preschool Child, Premature Birth, Language Tests, Language Development, Risk Factors

## Abstract

**Purpose:**

to verify if the performance of pre-school children born prematurely and at term in the Bayley-III language subtest differs and to identify whether variables gestational age, birth weight, socioeconomic level, and maternal education are determinant in the outcome of language development.

**Methods:**

Descriptive cross-sectional case-control study in which 36 pre-school children born prematurely and 27 born at term were evaluated concerning language development by the Bayley III subtest. Preschoolers between 18 and 36 months of chronological age were considered; with no syndromes or genetic, sensory, neurological, auditory, or visual impairments; and had not previously undergone speech therapy. Mann-Whitney, Fisher's Exact, and binary logistic regression tests were used for statistical analysis.

**Results:**

the groups' performance did not differ either by the composite score (p = .701) or by the classification based on the percentile (p = .225). Gestational age, birth weight, and socioeconomic status did not influence the outcome of language development. However, maternal education was significant (p = .014) in the binary logistic regression model, suggesting that the mother having studied until basic education increases the chance of having a child underperforming in the Bayley III language subtest 6.31 times.

**Conclusion:**

there was no difference between the groups in the Bayley-III language subtest and only maternal education influenced the outcome of language development.

## INTRODUCTION

Premature birth, which occurs before 37 weeks of gestation, increases the risk of long-term medical complications and compromised neurological development, in addition to family stress and social cost ^(1)^.

Prematurity has also been associated with a higher risk of impairment in cognitive and language development ^(2,3)^. This scenario is aggravated when associated with preterm birth, socio-environmental factors (low level of maternal education and quality of the family environment) or biomedical (male gender, Apgar score, birth weight, length of stay in the neonatal intensive care unit (NICU) and medical complications such as bronchopulmonary dysplasia, intraventricular hemorrhage, and neonatal sepsis) ^(4–6)^.

About 6.9% and 29.3% of children born prematurely present cognitive and language impairments, respectively ^(7)^, with a higher probability of language impairment between 2 and 4 years old ^(8)^. Furthermore, the difficulties resulting from prematurity can extend throughout life, interfering with school-age performance, compromising reading, spelling, math, and memory skills ^(9,10)^.

Thus, the surveillance of the development of children born prematurely is essential to identify developmental deviations and detect specific needs of each child, to propose the best intervention ^(3)^.

The Bayley Infant and Young Child Development Scale is the instrument internationally considered as the gold standard for cognitive and language assessment in preschoolers. Currently, in its third edition, Bayley III presents five distinct subtests for assessing child development in the areas of cognition, language, motor, socio-emotional and adaptive behavior ^(11)^. As this is a complete assessment battery, its average administration time is ninety minutes, depending on both the child's collaboration and behavior, as well as on the evaluator's ability.

In Brazil, although unofficial translations of the instrument already existed, they were used mainly in research contexts ^(12)^. In recent years, a study was carried out to translate and adapt the instrument to provide evidence of the convergent validity and reliability of the Brazilian version in children aged 12 to 42 months in the Southeast region. However, there are still no reference standards for Brazilian culture ^(13)^. In 2018, however, this instrument was commercially released in the country.

Among the five areas evaluated, the highest frequency of developmental impairments in premature children has been described in the language ^(7)^. The Bayley III scale is more advantageous to assess language separately, discriminating between receptive and expressive language impairments ^(14)^. This subtest includes receptive and expressive communication skills and provides information on understanding and responding to verbal stimuli, vocalization, naming, and communicative ability with others. Despite this, few studies have used the language subtest after its commercialization to verify its performance in the assessment of language development in the Brazilian context ^(15)^.

Considering the cultural and linguistic particularities involved in communication and that children born prematurely are usually among those that most demand developmental assessment, the objectives of this study were (1) to verify whether the performance of preschool children born preterm and at term in the subtest of Bayley-III language differs and (2) identify whether gestational age, birth weight, socioeconomic level, and maternal education variables are determinant in the outcome of language development.

## METHODS

This is a descriptive cross-sectional case-control study, approved by the Research Ethics Committee of Onofre Lopes University Hospital (CEP HUOL) at “Universidade Federal do Rio Grande do Norte” (UFRN), (CAAE: 97759718.4.0000.5292).

The groups were composed of preschool children born preterm (<37 gestational weeks) and at term treated at two childcare clinics of the Department of Pediatrics of the University Hospital. For eligibility criteria for both groups, the chronological age between 18 and 36 months was considered; absence of syndromes or genetic, sensory, neurological, auditory, or visual impairments, and not having undergone speech-language therapy. The presence of a family or medical complaint related to language development was not considered an exclusion criterion.

The selection of subjects was performed by consulting the medical record, considering gestational age and birth weight. In the case of preterm infants, the clinical condition (intracranial hemorrhage grades I and II, neonatal sepsis, bronchopulmonary dysplasia) and length of stay in the NICU were also considered.

Next, those responsible were contacted and invited to participate in the study after being informed about its conduct. Those who agreed signed an informed consent form and participated in a brief interview to obtain information on the preschool's general development, family history of speech-language disorders, and environmental factors. The characterization of the socioeconomic level of the participants was based on the mother's level of education and the classification of the family according to Critério Brasil ^(16)^. Each of these variables was grouped into two different categories for better comparison of results: maternal education was categorized into basic education or higher education; and the socio-economic classification of the family was categorized as “D-E or C2” and “C1, B2 or B1”.

The premature group (PG) was composed of 36 preschool children, 18 (50%) of which were male. Regarding complications associated with preterm birth, 22.2% had bronchopulmonary dysplasia; 41.7% early, late, or both neonatal sepsis; 25% had grade I intracranial hemorrhage and 5.6% grade II; and 66.7% remained hospitalized in the neonatal ICU for more than 15 days after birth. The term group (TG) was composed of 27 preschool children, 19 (70.4%) males. The groups differed only by gestational age and birth weight (Table 1).

**Table 1:** Characterization of premature and term-born groups

Variable	Description	Premature Group			Term Group			p
		Average	SD	Interval	Average	SD	Interval	
Age at collection	Months	26	5.14	18 - 35	27	4.91	18 - 35	0.233
Maternal age	Years	29	6.34	18 - 42	30	6.26	19 - 41	0.854
Gestational age	Weeks	30	2.77	25 - 35	40	1.49	37 - 42	<0.001*
Weight	Birth weight	1305,1	402.81	645 - 2208	3314.9	405.65	2470 - 4200	<0.001*
		n	%		n	%		
Sex	Male	18	50.0%		19	70.4%		0.104
	Female	18	50.0%		8	29.6%		
Type of birth	Normal	17	47.2%		9	33.3%		0.268
	Caesarean	19	52.3%		18	66.7%		
Maternal education	Basic education	33	91.7%		21	77.8%		0.119
	College education	3	8.3%		6	22.2%		
Socioeconomic level	D-E or C2	26	72.2%		20	74.0%		0.870
	C1, B2 or B1	10	27.8%		7	26.0%		

**Caption:** SD: standard deviation; range: displays the minimum and maximum value; n: number of subjects; %: percentage of subjects in the sample

* Statistical difference (p=0.05) – Mann-Whitney test

The Bayley III language subtest was applied and analyzed according to the instrument's instructions, and percentile scores were adopted for the outcome (performance) of language development with a 95% confidence interval. It is worth emphasizing that for preschool children born prematurely, up to 24 months of age, the age was corrected, as provided for in the instrument. The variables "composite score" and "percentile classification" were considered for the analysis of results, considering the grouping of percentile classification into "below average" and "average or above average".

Statistical analysis was performed using the Statistical Package for Social Sciences (SPSS) version 20 and the significance level adopted was 5%. Descriptive analyzes of the numerical variables that characterize the subjects were arranged through the mean, standard deviation, and range of minimum and maximum values. For categorical variables, the frequency of distribution and the equivalent percentage were used. To check whether there was a difference in any age group between the groups, in the comparison, it was decided to stratify the subjects according to age group.

To verify the data distribution, the Kolgomorov-Smirnov test was performed and it was found that the data distribution did not respect normality, therefore, the inferential analyzes were performed with non-parametric tests. The Mann-Whitney test compared the performance of groups in the language subtest and Fisher's exact test verified the association between the group and the classification of language performance. Finally, logistic regression was performed using the likelihood ratio method to verify the influence of gestational age, birth weight, socioeconomic level, and maternal education on performance in the language subtest.

## RESULTS

When comparing the composite score in the language subtest, there was no statistical difference between the groups, even when the subjects were regrouped according to their age group (Table 2). Considering the classification based on the percentile of the language subtest (outcome), there was also no difference between the groups (Table 3).

**Table 2:** Comparison of composite language subtest scores between groups

Age group	n	Group	Median	Interquartile range		p
18-24 months	17	Premature	89.0	76.5	94.0	0.949
	7	Term	91.0	68.0	97.0	
25-30 months	10	Premature	94.0	90.5	100.8	0.691
	12	Term	92.5	75.3	105.3	
31-36 months	9	Premature	91.0	87.5	98.5	0.663
	8	Term	97.0	79.3	104.5	
General	36	Premature	91.0	86.0	99.3	0.701
	27	Term	91.0	77.0	103.0	

**Caption:** n: number of subjects; p = statistical significance of the nonparametric Mann-Whitney test

**Table 3:** Comparison of language performance outcome

Age group	Outcome	Group		Total	p
		Premature	Term		
18-24 months	Below average	14 (82.3%)	6 (85.7%)	20	0.672
	average or above average	3 (17.7%)	1 (14.3%)	4	
25-30 months	Below average	6 (60.0%)	7 (58.3%)	13	0.639
	average or above average	4 (40.0%)	5 (41.7%)	9	
31-36 months	Below average	7 (77.8%)	4 (50.0%)	11	0.247
	average or above average	2 (22.2%)	4 (50.0%)	6	
General	Below average	27 (75.0%)	17 (63.0%)	44	0.225
	average or above average	9 (25.0%)	10 (37.0%)	19	

**Caption:** p = statistical significance of Fisher's exact test

Binary logistic regression considered the outcome in the language subtest as a dependent variable, and gestational age and birth weight as continuous independent variables, and the family's socioeconomic level and maternal education as categorical variables. Only the model containing maternal education was significant [X^2^ (1) = 6.072; p = 0.014, R^2^_Negelkerke_ = 0.130]. The mother having studied up to basic education was a significant predictor (OR = 6.31; 95% CI = 1.38 – 28.84), while gestational age, birth weight, and socioeconomic status were not.

## DISCUSSION

The present study compared the outcome of language performance on the Bayley III scale in preterm and born preschool children, in addition to investigating the influence of gestational age, birth weight, socioeconomic level, and maternal education on this outcome.

There was no statistical difference in language performance assessed by Bayley III between preterm and full-term infants, that is, the scale does not differentiate between preterm and full-term preschool children. Both groups had similar outcomes and both variables considered (compound score and percentile classification) were not statistically different.

This lack of distinction between groups can be interpreted in three ways: the first may indicate that in this age group, language development would not be influenced by the moment of birth (premature x term); the second may indicate that, due to the lack of validation of the instrument in Brazil, it is not sensitive and specific to identify typical and atypical patterns of language development in this population; and the third, on the contrary, may be related to the fact that both groups are composed of subjects with performance as expected or below expectations.

The first hypothesis coincides with the findings found in other studies ^(17,18)^ in which no significant differences were identified at two years of age in linguistic development between preterm and full-term children, preterm children tended to naturally recover primary acquisitions during the second year of life. Although the literature also reports losses in this age group ^(19,20)^, there is no consensus on the impact of prematurity on pre-linguistic communicative development until the end of the first year of age ^(21)^.

However, the authors of a meta-analysis reported increased difficulties in language tests in children born preterm from 3 years old ^(22)^. This variety of findings could be explained by the different methods, study designs, and sample characteristics used in research ^(23)^.

As our study covers preschoolers aged between 18 and 36 months, we chose not to consider only the performance of the groups as a whole, but to stratify them exactly to understand whether there would be distinct patterns before and after 24 months of chronological age. However, none of these age groups showed a different pattern between preterm and full-term.

Regarding the second possibility, it is important to highlight that a validation study was carried out in Brazil, but in only one region of Brazil, and the values ​​for the region were not specified. Thus, these authors reinforce the need for studies involving samples from different regions of the country, as well as longitudinal data to establish development curves comparing performance in different age groups ^(13)^.

A crucial aspect to be considered when dealing with language refers to the particularities of the language. Bayley III was designed and standardized in English, thus, considering the linguistic and cultural differences between English and Portuguese, several factors could influence the assessment and, consequently, the outcome. Differences in the use of the plural, verb tenses, and pronouns, as well as the structuring of sentences are examples of linguistic aspects that can interfere with the result, including difficulties in understanding the items ^(24)^. A possible strategy to clarify this interference would be to analyze the differences between the receptive and expressive subtests, but it would be necessary to consider item by item in each area, which would not fit in this initial study.

The third hypothesis, which considers that both groups would have subjects performing as expected or below expectations, demands a more careful analysis of the outcome. It is worth remembering that the subjects that make up both groups were selected regardless of complaints related to language development. Thus, it is noteworthy that, regardless of the general or stratified analysis, most subjects performed below average.

This finding leads us to consider that the absence of difference between the groups may result from the fact that these subjects share similar language skills and difficulties. That is, regardless of the moment of birth, they are immersed in an environment that similarly stimulates them. Therefore, in order to advance in understanding these aspects, we have to consider the analysis of determining variables.

Regarding the influence of the independent variables analyzed in the classification of the performance of the language subtest, the model applied in the binary logistic regression showed that the variables “birth weight”, “gestational age” and “socioeconomic level” do not interfere with its outcome.

For this sample, biological variables (gestational age and birth weight) did not influence the outcome, however, previous studies with a population born prematurely suggest that both represent the greatest risks for developmental impairments ^(25,26)^. The divergence of our findings may even result from the lack of studies to verify the sensitivity and specificity of Bayley III in Brazil, which may have interfered with the result, as mentioned above.

The absence of influence of socioeconomic level, on the other hand, may suggest that the measure used for this classification is not the most adequate to measure its influence on our reality. Critério Brasil takes into account, for the most part, the "buying" power of the population and their access to basic services, such as paving and sewage ^(16)^, without considering income and other relevant elements, such as the stimulation environment at home.

It is worth pointing out that studies that evaluated the language development of preschoolers in a situation of economic vulnerability (low income) found positive effects on language performance associated with the environment of stimulation at home and parents' education ^(27,28)^.

Accordingly, our findings showed the influence of the environmental variable “maternal education” on language performance. Particularly interesting, the effect of maternal education reached a significant level (OR = 6.31) which suggests that the mother having studied less (until basic education) increases the risk for underperformance on the Bayley III language subtest in preschoolers ^(18)^. Thus, this variable could be considered more sensitive in the assessment of language development to perform analyzes and comparisons.

When considering all analyzes of linguistic performance based on Bayley III, it is possible to affirm that in this population, the environment seems to have a more marked impact on development than aspects related to birth. Returning to the discussion of the hypotheses that would explain the absence of difference between preterm and term, we note that both socioeconomic vulnerabilities may interfere with the full development of communication skills, and Bayley III may not be able to analyze particularities of Brazilian culture penalizing this population. Thus, to better clarify this scenario, it is essential to expand the sample, especially for subjects born at term, and analyze the specific items that make up the instrument.

As limitations of this study, we found that most of the sample is composed of participants in socioeconomic vulnerability situations (classes DE and C2) and with a predominance of maternal education in basic education, the lack of another assessment instrument that could compare the findings with those of the Bayley III scale in this age group, as well as a longitudinal follow-up of the subjects, which would allow the interpretation of the findings as a cause in the outcome and greater control of the variables. We still consider the disparity between the number of subjects a limiting factor, however, it was quite challenging to convince parents of full-term preschoolers to participate in the study, especially before two years of life, since in the absence of complaints related to their language motivation was negligible.

However, the study brings important reflections on the contribution of biological and environmental aspects to the surveillance of early childhood development. From the research point of view, it contributes to the debate regarding the impact of vulnerability on linguistic development, signaling the need to discuss the measures used to measure environmental aspects, as well as the importance of having validated instruments. From a clinical point of view, it points to the need for careful analysis in the selection and application of the assessment instrument, regardless of its commercial availability, since the diagnosis requires a broader understanding of its development.

## CONCLUSION

There was no statistical difference between the performance of preterm and full-term preschool children on the Bayley-III language subtest. In this study, birth weight, gestational age, and socio-economic level did not influence the outcome of their language development; however, maternal education influenced, suggesting that having studied only until basic education increases the chance of having a child with a performance below expectations in the Bayley III language subtest by 6.31 times.
