# Supplementary material for: What factors interfere with the performance of preschool children in the language subtest of Bayley-III?
Source: Codas. 2021 Nov 1;34(1):e20200200. doi: 10.1590/2317-1782/20212020200 (PMC9886127; doi:10.1590/2317-1782/20212020200)
Supplement: Translation

# Translation: Quais fatores interferem no desempenho de pré-escolares no subteste de linguagem da Bayley-III?

**Authors:** Antonio Marcos Oliveira de Lima, Ana Manhani Cáceres-Assenço

**Affiliations:** 1 Laboratório de Desenvolvimento da Linguagem, Departamento de Fonoaudiologia, Centro de Ciências da Saúde, Universidade Federal do Rio Grande do Norte – UFRN - Natal (RN), Brasil.

**Keywords:** Pré-Escolares, Nascimento Prematuro, Testes de Linguagem, Desenvolvimento da Linguagem, Fatores de Risco

## Abstract

**Objetivo:**

verificar se o desempenho de pré-escolares nascidos prematuros e a termo no subteste de linguagem da Bayley-III difere e identificar se as variáveis idade gestacional, peso ao nascer, nível socioeconômico e escolaridade materna são determinantes no desfecho de desenvolvimento de linguagem.

**Método:**

estudo transversal descritivo caso controle em que 36 pré-escolares nascidos prematuros e 27 nascidos a termo foram avaliados em relação ao desenvolvimento de linguagem pelo subteste da Bayley III. Foram considerados pré-escolares entre 18 e 36 meses de idade cronológica; com ausência de síndromes ou alterações genéticas, sensoriais, neurológicas, auditivas ou visuais; e que não tinham realizado terapia fonoaudiológica previamente. Os testes de Mann-Whitney, Exato de Fisher e regressão logística binária foram utilizados para análise estatística.

**Resultados:**

o desempenho dos grupos não diferiu seja pela pontuação composta (p=0,701) ou pela classificação baseada no percentil (p=0,225). A idade gestacional, o peso ao nascimento e o nível socioeconômico não influenciaram no desfecho do desenvolvimento de linguagem. No entanto, a escolaridade materna foi significativa (p=0,014) no modelo de regressão logística binária, sugerindo que a mãe ter estudado até a educação básica aumenta a chance de ter um filho com desempenho abaixo do esperado no subteste de linguagem da Bayley III em 6,31 vezes.

**Conclusão:**

não houve diferença entre os grupos no subteste de linguagem da Bayley-III e apenas a escolaridade materna influenciou no desfecho do desenvolvimento de linguagem.

## INTRODUÇÃO

O nascimento prematuro, ocorrido antes das 37 semanas de gestação, aumenta o risco de complicações médicas a longo prazo e de comprometimento do desenvolvimento neurológico, além do estresse familiar e do custo social ^(1)^.

A prematuridade também tem sido associada a mais risco de alterações no desenvolvimento cognitivo e de linguagem ^(2,3)^. Esse cenário se agrava quando associado ao parto prematuro há fatores socioambientais (baixo nível de escolaridade materna e qualidade do ambiente familiar) ou biomédicos (sexo masculino, pontuação do Apgar, peso ao nascer, tempo de permanência na unidade de terapia intensiva neonatal (UTIN) e complicações médicas como a displasia broncopulmonar, hemorragia intraventricular e sepse neonatal) ^(4 ^–^ 6)^.

Cerca de 6,9% e 29,3% das crianças nascidas prematuras apresentam alterações cognitivas e de linguagem, respectivamente ^(7)^, com maior probabilidade de alteração na linguagem entre 2 e 4 anos ^(8)^. Além disso, as dificuldades em decorrência da prematuridade podem se estender ao longo da vida, interferindo no desempenho em idade escolar, com comprometimento de habilidades de leitura, soletração, matemática e memória ^(9,10)^.

Assim, a vigilância do desenvolvimento de crianças nascidas prematuras é essencial para identificar desvios no desenvolvimento e detectar necessidades específicas de cada criança, com a finalidade de propor a melhor intervenção ^(3)^.

O instrumento internacionalmente considerado padrão-ouro para avaliação cognitiva e de linguagem em pré-escolares é a escala Bayley de desenvolvimento do bebê e da criança pequena. Atualmente em sua terceira edição, a Bayley III apresenta cinco subtestes distintos para avaliação do desenvolvimento infantil nas áreas de cognição, linguagem, motor, socioemocional e comportamento adaptativo ^(11)^. Por se tratar de uma bateria completa de avaliação, seu tempo médio de administração é de noventa minutos, dependendo tanto da colaboração e do comportamento da criança, quanto da habilidade do avaliador.

No Brasil, embora já existissem traduções não oficiais do instrumento, essas eram utilizadas principalmente em contextos de pesquisa ^(12)^. Nos últimos anos, foi realizado um estudo para traduzir e adaptar o instrumento para fornecer evidências da validade e confiabilidade convergentes da versão brasileira em crianças de 12 a 42 meses na região sudeste, no entanto ainda não há normas de referência para a cultura brasileira ^(13)^. Em 2018, entretanto, este instrumento passou a ser comercializado no país.

Dentre as cinco áreas avaliadas, a maior frequência de alterações no desenvolvimento em crianças prematuras tem sido descrita na linguagem ^(7)^. A escala Bayley III tem se mostrado mais vantajosa para avaliar linguagem separadamente, discriminando prejuízos entre a linguagem receptiva e expressiva ^(14)^. Este subteste inclui habilidades de comunicação receptiva e expressiva e fornece informações sobre a compreensão e a resposta a estímulos verbais, à vocalização, nomeação e habilidade comunicativa com outras pessoas. Apesar disso, poucos estudos têm utilizado o subteste de linguagem após sua comercialização para verificar seu desempenho na avaliação do desenvolvimento de linguagem no contexto brasileiro ^(15)^.

Considerando as particularidades culturais e linguísticas envolvidas na comunicação e que as crianças nascidas prematuras costumam estar entre as que mais demandam avaliação do desenvolvimento, os objetivos desse estudo foram (1) verificar se o desempenho de pré-escolares nascidos prematuros e a termo no subteste de linguagem da Bayley-III difere e (2) identificar se as variáveis idade gestacional, peso ao nascer, nível socioeconômico e escolaridade materna são determinantes no desfecho de desenvolvimento de linguagem.

## MÉTODO

Trata-se de um estudo transversal descritivo caso controle, aprovado pelo Comitê de Ética em Pesquisa do Hospital Universitário Onofre Lopes (CEP HUOL) da Universidade Federal do Rio Grande do Norte (UFRN), (CAAE: 97759718.4.0000.5292).

Os grupos foram compostos por pré-escolares nascidos prematuros (<37 semanas gestacionais) e a termo atendidos em dois ambulatórios de puericultura do Departamento de Pediatria do Hospital Universitário. Para critérios de elegibilidade de ambos os grupos foi considerada a idade cronológica entre 18 e 36 meses de idade; ausência de síndromes ou alterações genéticas, sensoriais, neurológicas, auditivas ou visuais, e não ter realizado terapia fonoaudiológica. A presença de queixa familiar ou médica relacionada ao desenvolvimento da linguagem não foi considerada como critério de exclusão.

A seleção dos sujeitos foi realizada por meio de consulta ao prontuário médico considerando idade gestacional e peso ao nascer. No caso dos nascidos prematuros, foi considerado também o quadro clínico (hemorragia intracraniana com graus I e II, sepse neonatal, displasia broncopulmonar) e o tempo de internação em UTIN.

A seguir os responsáveis foram contatados e convidados a participar do estudo após serem esclarecidos sobre sua condução. Aqueles que concordaram, assinaram o termo de consentimento livre e esclarecido e participaram de uma breve entrevista para obter informações do desenvolvimento geral do pré-escolar, histórico familiar de alteração de fala/linguagem, e fatores ambientais. A caracterização do nível socioeconômico dos participantes foi baseada no nível de escolaridade da mãe e na classificação da família conforme o Critério Brasil ^(16)^. Cada uma dessas variáveis foi agrupada em duas categorias diferentes para melhor comparação dos resultados: a escolaridade materna foi categorizada em educação básica ou educação superior; e a classificação socioeconômica da família foi categorizada em “D-E ou C2” e “C1, B2 ou B1”.

O grupo prematuro (GP) foi composto por 36 pré-escolares, sendo 18 (50%) do sexo masculino. Em relação às complicações associadas ao parto prematuro, 22,2% apresentaram displasia broncopulmonar; 41,7% sepse neonatal precoce, tardia ou ambas; 25% apresentaram hemorragia intracraniana grau I e 5,6% grau II; e 66,7% permaneceram internados em UTI neonatal por mais de 15 dias após o nascimento. O grupo termo (GT) foi composto por 27 pré-escolares, sendo 19 (70,4%) do sexo masculino. Os grupos diferiram apenas pela idade gestacional e pelo peso ao nascer (Tabela 1).

**Tabela 1:** Caracterização dos grupos prematuros e nascidos a termo

Variável	Descrição	Grupo Prematuro			Grupo Termo			p
		média	DP	Intervalo	média	DP	Intervalo	
Idade na coleta	Meses	26	5,14	18 - 35	27	4,91	18 - 35	0,233
Idade materna	Anos	29	6,34	18 - 42	30	6,26	19 - 41	0,854
Idade gestacional	Semanas	30	2,77	25 - 35	40	1,49	37 - 42	<0,001*
Peso	Peso ao nascer	1305,1	402,81	645 - 2208	3314,9	405,65	2470 - 4200	<0,001*
		n	%		n	%		
Sexo	Masculino	18	50,0%		19	70,4%		0,104
	Feminino	18	50,0%		8	29,6%		
Tipo de parto	Normal	17	47,2%		9	33,3%		0,268
	Cesariana	19	52,3%		18	66,7%		
Escolaridade materna	Educação básica	33	91,7%		21	77,8%		0,119
	Educação superior	3	8,3%		6	22,2%		
Nível socioeconômico	D-E ou C2	26	72,2%		20	74,0%		0,870
	C1, B2 ou B1	10	27,8%		7	26,0%		

**Legenda:** DP: desvio-padrão; intervalo: apresenta o valor mínimo e o máximo; n: número de sujeitos; %: porcentagem de sujeitos na amostra

* diferença estatística (p=0,05) – teste não paramétrico de Mann-Whitney

O subteste de linguagem da Bayley III foi aplicado e analisado conforme as instruções do instrumento e a pontuação dos percentis foi adotada para o desfecho (desempenho) do desenvolvimento de linguagem com intervalo de confiança de 95%. Vale reforçar que para os pré-escolares nascidos prematuros, até 24 meses a idade foi corrigida, como previsto no instrumento. As variáveis “pontuação composta” e “classificação em percentil” foram consideradas para a análise dos resultados, considerando o agrupamento da classificação em percentis em “abaixo da média” e “média ou acima da média”.

A análise estatística foi realizada no *Software Statistical Package for the Social Sciences (SPSS)* versão 20 e o nível de significância adotado foi de 5%. As análises descritivas das variáveis numéricas que caracterizam os sujeitos foram dispostas por meio da média, desvio-padrão e intervalo dos valores mínimo e máximo. Para as variáveis categóricas foram utilizadas a frequência de distribuição e a percentagem equivalente. Para verificar se existia diferença em alguma faixa etária entre os grupos, na comparação optou-se por estratificar os sujeitos de acordo com a faixa etária.

Para verificar a distribuição dos dados foi realizado o teste de Kolgomorov-Smirnov e verificou-se que a distribuição dos dados não respeitava a normalidade, portanto as análises inferenciais foram realizadas com testes não paramétricos. O teste de Mann-Whitney comparou o desempenho dos grupos no subteste de linguagem e o teste exato de Fisher verificou a associação entre o grupo e a classificação do desempenho em linguagem. Por fim, foi realizada regressão logística pelo método de razão de verossimilhança para verificar a influência da idade gestacional, peso ao nascer, nível socioeconômico e escolaridade materna no desempenho no subteste de linguagem.

## RESULTADOS

Ao comparar a pontuação composta no subteste de linguagem não houve diferença estatística entre os grupos, mesmo quando os sujeitos foram reagrupados de acordo com sua faixa etária (Tabela 2). Considerando a classificação baseada no percentil do subteste de linguagem (desfecho final) também não houve diferença entre os grupos (Tabela 3).

**Tabela 2:** Comparação da pontuação composta do subteste de linguagem entre os grupos

Faixa etária	n	Grupo	Mediana	Intervalo interquartil		p
18-24 meses	17	Prematuro	89,0	76,5	94,0	0,949
	7	Termo	91,0	68,0	97,0	
25-30 meses	10	Prematuro	94,0	90,5	100,8	0,691
	12	Termo	92,5	75,3	105,3	
31-36 meses	9	Prematuro	91,0	87,5	98,5	0,663
	8	Termo	97,0	79,3	104,5	
Geral	36	Prematuro	91,0	86,0	99,3	0,701
	27	Termo	91,0	77,0	103,0	

**Legenda:** n: número de sujeitos; p = significância estatística do teste não paramétrico de Mann-Whitney

**Tabela 3:** Comparação do desfecho do desempenho de linguagem

Faixa etária	Desfecho	Grupo		Total	p
		Prematuro	Termo		
18-24 meses	Abaixo da média	14 (82,3%)	6 (85,7%)	20	0,672
	Média ou acima da média	3 (17,7%)	1 (14,3%)	4	
25-30 meses	Abaixo da média	6 (60,0%)	7 (58,3%)	13	0,639
	Média ou acima da média	4 (40,0%)	5 (41,7%)	9	
31-36 meses	Abaixo da média	7 (77,8%)	4 (50,0%)	11	0,247
	Média ou acima da média	2 (22,2%)	4 (50,0%)	6	
Geral	Abaixo da média	27 (75,0%)	17 (63,0%)	44	0,225
	Média ou acima da média	9 (25,0%)	10 (37,0%)	19	

**Legenda:** p = significância estatística do teste exato de Fisher

A regressão logística binária considerou como variável dependente o desfecho no subteste de linguagem e como variáveis independentes contínuas a idade gestacional e o peso ao nascer, e categóricas o nível socioeconômico da família e escolaridade materna. Apenas o modelo contendo a escolaridade materna foi significativo [X^2^ (1) = 6,072; p = 0,014, R^2^_Negelkerke_ = 0,130]. A mãe ter estudado até a educação básica foi um previsor significativo (OR = 6,31; IC 95% = 1,38 – 28,84), enquanto a idade gestacional, o peso ao nascimento e o nível socioeconômico não foram.

## DISCUSSÃO

O presente estudo comparou o desfecho do desempenho de linguagem da escala Bayley III em pré-escolares prematuros e nascidos, além de investigar a influência da idade gestacional, peso ao nascer, nível socioeconômico e escolaridade na mãe neste desfecho.

Não houve diferença estatística no desempenho de linguagem avaliado pela Bayley III entre nascidos prematuros e a termo, ou seja, a escala não diferencia os pré-escolares prematuros dos nascidos a termo. Ambos os grupos apresentaram desfechos similares e ambas as variáveis consideradas (pontuação composta e classificação em percentil) não apresentaram diferença estatística.

Essa ausência de distinção entre os grupos pode ser interpretada de três formas: a primeira pode indicar que nesta faixa etária o desenvolvimento da linguagem realmente não seria influenciado pelo momento do nascimento (prematuro x termo); a segunda pode indicar que pela ausência de validação do instrumento no Brasil ele não seja sensível e específico para identificar padrões típicos e atípicos do desenvolvimento da linguagem nesta população; e a terceira, contrariamente, pode estar relacionada ao fato de ambos os grupos serem compostos por sujeitos com desempenho dentro do esperado quanto abaixo do esperado.

A primeira hipótese coincide com os achados encontrados em outros estudos ^(17,18)^ em que não foram identificadas diferenças significativas aos dois anos de idade no desenvolvimento linguístico entre crianças prematuras e a termo, as crianças prematuras apresentaram tendência a recuperar naturalmente as aquisições primárias durante o segundo ano de vida. Embora a literatura também relate prejuízos nessa faixa de idade ^(19,20)^, não há consenso sobre o impacto da prematuridade no desenvolvimento comunicativo pré-linguístico até o final do primeiro ano de idade ^(21)^.

No entanto, os autores de uma meta-análise relataram aumento das dificuldades nos testes de linguagem em crianças nascidas prematuras a partir dos 3 anos de idade ^(22)^. Essa variedade de achados poderia ser explicada pelos diferentes métodos, desenhos de estudo e características da amostra utilizados nas pesquisas ^(23)^.

Como nosso estudo abrange pré-escolares entre 18 e 36 meses, optamos por não considerar apenas o desempenho dos grupos como um todo, mas estratificá-los exatamente para compreender se haveriam padrões distintos antes e após os 24 meses de idade cronológica. Entretanto, não houve nenhuma destas faixas etárias que tenha apresentado um padrão diferente entre prematuros e nascidos a termo.

Com relação à segunda possibilidade, é importante destacar que um estudo de validação foi realizado no Brasil, porém em apenas uma região do Brasil e não foram especificados os valores para a região. Desse modo, estes autores reforçam a necessidade de estudos envolvendo amostras de diferentes regiões do país, bem como dados longitudinais para estabelecer curvas de desenvolvimento comparando o desempenho em diferentes faixas etárias ^(13)^.

Um aspecto crucial para ser considerado ao tratarmos de linguagem se refere as particularidades da língua. A Bayley III foi construída e normatizada na língua inglesa, assim, considerando as diferenças linguísticas e culturais entre o inglês e o português, vários fatores poderiam influenciar na avaliação e, consequentemente, no desfecho. As diferenças no uso do plural, de tempos verbais e pronomes, bem como a estruturação de frases são exemplos de aspectos linguísticos que podem interferir no resultado, incluindo dificuldades na compreensão dos itens ^(24)^. Uma possível estratégia para esclarecer essa interferência seria analisar as diferenças entre os subtestes receptivo e expressivo, porém seria necessário considerar item a item de cada área, o que não caberia neste estudo inicial.

A terceira hipótese, que considera que ambos os grupos teriam sujeitos com desempenho dentro do esperado quanto abaixo do esperado, nos demanda uma análise mais atenta do desfecho. Vale lembrar que os sujeitos que compõem ambos os grupos foram selecionados independente de queixa relacionada ao desenvolvimento da linguagem. Assim, nos chama atenção que, independente da análise geral ou estratificada, a maioria dos sujeitos teve desempenho abaixo da média.

Tal constatação nos leva a considerar que é possível que a ausência de diferença entre os grupos possa decorrer do fato de que estes sujeitos compartilham habilidades e dificuldades de linguagem semelhantes. Ou seja, independente do momento de nascimento, eles estão imersos em um ambiente que os estimula de forma similar. Logo, para avançarmos na compreensão destes aspectos temos que considerar a análise das variáveis determinantes.

Em relação à influência das variáveis independentes analisadas na classificação do desempenho do subteste de linguagem, o modelo aplicado na regressão logística binária mostrou que as variáveis “peso ao nascer”, “idade gestacional” e “nível socioeconômico” não interferem em seu desfecho.

Para esta amostra, as variáveis biológicas (idade gestacional e peso ao nascer) não influenciaram o desfecho, entretanto, estudos prévios com população nascida prematura sugerem que ambas representam os maiores riscos para alterações no desenvolvimento ^(25,26)^. A divergência dos nossos achados pode inclusive decorrer da falta de estudos para verificar a sensibilidade e especificidade da Bayley III no Brasil, que pode ter interferido no resultado, como mencionado anteriormente.

A ausência de influência do nível socioeconômico, por outro lado, pode sugerir que a medida utilizada para esta classificação não seja a mais adequada para mensurar sua influência na nossa realidade. O Critério Brasil leva em consideração, majoritariamente, o poder de “compra” da população e seu acesso a serviços básicos, como pavimentação e esgoto (16), sem considerar a renda e outros elementos relevantes, como por exemplo o ambiente de estimulação de casa.

Vale pontuar que estudos que avaliaram o desenvolvimento da linguagem de pré-escolares em situação de vulnerabilidade econômica (baixa renda) encontraram efeitos positivos no desempenho de linguagem associados ao ambiente de estimulação de casa e educação dos pais ^(27,28)^.

Em consonância, nossos achados mostraram que influência da variável ambiental “escolaridade materna” no desempenho da linguagem. Particularmente interessante, o efeito da educação materna atingiu um nível significativo (OR = 6,31) que sugere que a mãe ter estudado menos (até a educação básica) aumenta o risco para o desempenho abaixo do esperado no subteste de linguagem da Bayley III em pré-escolares ^(18)^. Desse modo, tal variável poderia ser considerada mais sensível na avaliação do desenvolvimento de linguagem para realizar análises e comparações.

Ao considerarmos todas as análises do desempenho linguístico baseado na Bayley III é possível afirmar que nesta população o ambiente parece ter um impacto mais marcado no desenvolvimento do que os aspectos relacionados ao nascimento. Retomando a discussão das hipóteses que explicariam a ausência de diferença entre prematuros e termo, notamos que é possível que tanto a vulnerabilidade socioeconômica interfira no desenvolvimento pleno das habilidades comunicativas, quanto a Bayley III pode não ser capaz de analisar particularidades da cultura brasileira penalizando esta população. Assim, para melhor esclarecer este cenário é essencial que se amplie a amostra, especialmente de sujeitos nascidos a termo, e se analise os itens específicos que compõe o instrumento.

Este estudo apresenta como limitações o fato da maior parte da amostra ser composta por participantes em situação vulnerabilidade socioeconômica (classes D-E e C2) e com predomínio de escolaridade materna na educação básica, a falta de um outro instrumento de avaliação que pudesse comparar os achados com os da escala Bayley III nesta faixa etária, bem como um acompanhamento longitudinal dos sujeitos, que possibilitaria a interpretação dos achados como causa no desfecho e maior controle das variáveis. Consideramos ainda a disparidade entre o número de sujeitos um fator limitante, entretanto, foi bastante desafiador convencer pais de pré-escolares nascidos a termo a participar do estudo, especialmente antes dos dois anos de vida, visto que na ausência de queixas relacionadas à linguagem sua motivação era ínfima.

No entanto, o estudo traz importantes reflexões acerca da contribuição de aspectos biológicos e ambientais para a vigilância do desenvolvimento na primeira infância. Do ponto de vista da pesquisa, contribui para o debate referente ao impacto da vulnerabilidade no desenvolvimento linguístico, sinalizando a necessidade de discutirmos as medidas utilizadas para mensurar as aspectos ambientais, bem como a importância de termos instrumentos validados. Do ponto de vista clínico aponta a necessidade de uma análise criteriosa na seleção e aplicação do instrumento de avaliação, independente de sua disponibilidade comercial, visto que o diagnóstico demanda uma compreensão mais ampla do desenvolvimento.

## CONCLUSÃO

Não houve diferença estatística entre o desempenho de pré-escolares nascidos prematuros e a termo no subteste de linguagem da Bayley-III. Neste estudo, o peso ao nascer, a idade gestacional e o nível socioeconômico não influenciaram no desfecho de seu desenvolvimento de linguagem; porém, a escolaridade materna influenciou, sugerindo que a mãe ter estudado apenas até a educação básica aumenta a chance de ter um filho com desempenho abaixo do esperado no subteste de linguagem da Bayley III em 6,31 vezes.
